# Aiding Chronic Obstructive Pulmonary Disease and Congestive Heart Failure Ultrasound-Guided Management Through Enhanced Point-of-Care Ultrasound (ACCUMEN-POCUS): Protocol for a Randomized Controlled Trial

**DOI:** 10.2196/76186

**Published:** 2025-09-23

**Authors:** Michelle Nora Grinman, Peter Nakhla, Steve Reid, Dennis Moon, Negar Dehghan Noudeh, Oladoyin Olaosebikan, Amanda Chung Yan Ip, Salomé Saunders, Ryan Kozicky, John Conly, Andrew Wallace Kirkpatrick, Jeff Round, Irene Wai Yan Ma, Suean Pascoe, Ghazwan Altabbaa

**Affiliations:** 1 Department of Medicine Cumming School of Medicine University of Calgary Calgary Canada; 2 O’Brien Institute for Public Health Cumming School of Medicine University of Calgary Calgary, AB Canada; 3 Alberta Health Services Calgary, AB Canada; 4 Presuna Coleman, AB Canada; 5 Faculty of Medicine University of Toronto Toronto, ON Canada; 6 Snyder Institute for Chronic Diseases University of Calgary Calgary, AB Canada; 7 Department of Surgery Cumming School of Medicine University of Calgary Calgary Canada; 8 TeleMentored Ultrasound Supported Medical Interventions Research Group Calgary, AB Canada; 9 Faculty of Medicine and Dentistry University of Alberta Edmonton Canada; 10 Department of Community Health Sciences University of Calgary Calgary Canada; 11 Zedu Ultrasound Training Solutions Greensborough Australia

**Keywords:** hospital at home, point-of-care ultrasound, acute decompensated heart failure, ADHF, acute exacerbation of chronic obstructive pulmonary disease, AE-COPD, pneumonia

## Abstract

**Background:**

Hospital at home (HAH) programs offer acute care at home as a substitute for inpatient hospitalization, reducing health care costs while maintaining safety and care quality. Despite point-of-care ultrasound (POCUS) having been validated in inpatient and emergency settings, its role in HAH care remains underexplored. Common conditions treated in medical HAH programs, such as acute exacerbation of chronic obstructive pulmonary disease (AE-COPD), acute decompensated heart failure (ADHF), and pneumonia, are highly amenable to POCUS integration into clinical decision-making and have been proven to improve health care use outcomes. The portability of POCUS makes it ideal for use in HAH; however, its feasibility remains to be proven given the need for health care provider training and use in online settings.

**Objective:**

This study evaluates the feasibility and clinical utility of remotely interpreted lung and inferior vena cava (IVC) POCUS acquired by community paramedics to support real-time clinical decision-making for HAH patients with AE-COPD, ADHF, and pneumonia in Calgary, Alberta.

**Methods:**

This randomized controlled trial compares usual HAH care (control) to lung and IVC POCUS–enhanced HAH care (intervention). Handheld POCUS devices captured images that were securely shared using a cloud-based application. This enabled real-time image sharing among the clinical team, facilitating immediate decision-making by remote physicians. A mixed methods approach will evaluate clinical outcomes, patient experiences, health care use, and health care provider perceptions of POCUS integration. The primary outcome is defined as the length of stay for the index HAH admission. Quantitative analysis will assess clinical efficacy and health care resource use, while qualitative methods, such as interviews and surveys, will capture patient and health care provider experiences.

**Results:**

Study funding began in April 2022, and data collection commenced in December 2023. Patient recruitment was finalized on December 31, 2024. This study included a 3-month follow-up for significant outcomes and will include a 1-year follow-up for long-term health care use, including admissions to long-term care. In total, 20 patients were enrolled (intervention group: n=10, 50%; control group: n=10, 50%). Initial results highlighted the feasibility and potential benefits of remotely acquired POCUS imaging in HAH. Full data analysis is in progress.

**Conclusions:**

This study is the first randomized controlled trial to investigate remotely acquired POCUS by nonphysician practitioners for real-time lung and IVC remote decision-making in HAH care. Findings will provide insights into whether serial lung and IVC POCUS assessments improve ADHF, AE-COPD, and pneumonia outcomes in the HAH setting, enhancing understanding of the value of POCUS integration from a health care provider’s perspective. By assessing its clinical impact and feasibility, this research may inform future guidelines for incorporating POCUS into home-based care, ultimately improving patient care and optimizing health care resource use.

**Trial Registration:**

ClinicalTrials.gov NCT05423652; https://clinicaltrials.gov/study/NCT05423652

**International Registered Report Identifier (IRRID):**

DERR1-10.2196/76186

## Introduction

### Background

Acute decompensated heart failure (ADHF), acute exacerbation of chronic obstructive pulmonary disease (AE-COPD), and pneumonia are among the leading causes of patient morbidity and mortality worldwide. Patients with the aforementioned diseases present with dyspnea and respiratory distress [1,2]. Currently, diagnosis and management of these patients are reliant on symptoms, physical examination such as lung auscultation, radiographic imaging studies, and laboratory investigations [3-5]. However, for these conditions, the aforementioned modalities have poor diagnostic rates, with radiographs having false-negative rates exceeding 25% at times. Biomarkers have poor predictive value in patients with comorbidities presenting with multiple physiological derangements [6-8]. Furthermore, chest radiographs often require costly machinery housed in a hospital or radiology center, with the potential for delays due to radiologic interpretation time [9].

Emerging literature on using point-of-care ultrasound (POCUS) for management of AE-COPD, ADHF, and pneumonia shows reductions in hospital length of stay (LOS), favorable patient outcomes, and lowered health care costs [10-13]. These findings are often attributed to POCUS’s comparable or higher sensitivity relative to physical examination and chest radiography in detecting early signs of pulmonary edema, pleural effusions, impaired diaphragmatic excursion, and subpleural consolidation [14-16]. Unlike radiographic imaging, POCUS also offers advantages such as portability, absence of radiation exposure, and suitability for frequent bedside use in longitudinal care [15,17,18]. Guevarra and Greenstein [19] describe how POCUS is now integral to volume status assessment, cardiac function evaluation, and lung imaging in patients who are critically ill. Despite its clinical utility, widespread adoption of POCUS has historically been limited by the need for specialized training, a shortage of trained health care providers, and variability in image acquisition and interpretation [10,11,16].

While mobile health tools have gained traction in chronic disease management, relatively few have been designed specifically for acute cardiorespiratory conditions such as AE-COPD or ADHF. A systematic review of reviews found that mobile health interventions can improve symptoms and reduce hospitalizations in chronic pulmonary disease and heart failure, among other conditions [20]. However, most commercially available solutions focus on general wellness monitoring or outpatient management, with limited application to POCUS in acute care. Recent reviews have demonstrated that POCUS, particularly when integrated with mobile platforms, can substantially improve diagnostic accuracy and guide management in heart failure and other acute conditions [11,21]. This latter finding underscores the need for tailored, evidence-based tools that address specific diagnostic and triage challenges in these high-risk populations.

Recent studies suggested that nonexpert health care providers (and even patients themselves) could be effectively trained to acquire quality POCUS images in an acute care, emergency, or intensive care unit setting, particularly by using teleguidance methods [22-24]. Although POCUS is well established in emergency and critical care settings, its use has progressively expanded into community and home-based care environments [25-27]. This shift has been notably accelerated by the COVID-19 pandemic, which highlighted the need for accessible diagnostic modalities among homebound older adults and populations in geographically isolated regions. In this context, teleultrasound has emerged as a viable solution, supporting timely and accurate clinical decision-making within evolving models of home-based acute care [23,28-30].

This paper describes a study protocol developed to investigate the feasibility and clinical usefulness of embedding community paramedic (CP)–acquired lung and inferior vena cava (IVC) POCUS scans in supporting real-time remote decision-making by physicians of a hospital at home (HAH) program. The sharing of images, feedback, and clinical decision-making was supported by a cloud-based application (Presuna) designed to extract POCUS images from handheld POCUS devices to enable the sharing of ultrasound images among clinical team members for both educational and clinical care purposes.

### Objectives and Aims

This study is a multisite, 2-arm, parallel, randomized controlled trial (RCT) comparing enhanced daily assessments for patients with AE-COPD, ADHF, or pneumonia using POCUS with a cloud-based application versus standard care provided by an HAH program at a tertiary acute care medical teaching hospital in Calgary, Alberta.

Standard care provided through this HAH program was selected as the comparator to assess patient outcomes, experience, and health care use and to evaluate the economic feasibility of integrating POCUS into future standard care practices.

## Methods

### Study Setting

The Calgary Zone Virtual Home Hospital (previously the Complex Care Hub) [31] is an HAH program that provides substitute acute care in patients’ homes for a general internal medicine (GIM) population at 2 hospitals, the Rockyview General Hospital and South Health Campus. Patients can be admitted to HAH from a brick-and-mortar (BAM) hospital, either directly from the emergency department or the medical inpatient wards, as well as from community settings such as specialty clinics and family medicine referrals.

There are two collaborating teams:

1. The hospital-based team consists of a hospital physician (GIM or family medicine hospitalist), nurse navigators (NNs), and a pharmacist. This team performs case finding, in-person assessments, and interventions in a dedicated clinic space and oversees the care plan. In addition, physicians conduct online visits for patients admitted to the program, either directly leveraging digital remote patient monitoring technology or via an online consultation with home-visiting CPs.
2. The home-based team consists of highly skilled CPs who collaborate with patients and their caregivers in the home. CPs perform most home visits, assessments, and interventions under remote guidance by the HAH physician.

### Patient Recruitment

Patients admitted to HAH with ADHF, AE-COPD, or pneumonia were identified as potentially eligible either by HAH NNs or through the research team’s screening of the patient charts (under a waiver of consent). NNs or CPs offered eligible patients the opportunity to be contacted by the research team to learn about the research study. Following initial contact, consenting patients were contacted by research assistants for enrollment into the study, obtaining written or verbal informed consent via REDCap (Research Electronic Data Capture; Vanderbilt University) or email.

Inclusion criteria for patients were (1) a primary active diagnosis of ADHF, AE-COPD, or pneumonia requiring home-based acute care at the time of enrollment; (2) previous admission to the HAH program; and (3) willingness to participate in the study and provide informed consent. Patients were excluded from this study if they did not have 1 of the 3 index conditions, had new unstable rib fractures, a previous history of allergy to ultrasound gel, or chronic lung diseases that would confound POCUS imaging (pulmonary fibrosis, fibrothorax, lung cancer or other intrapulmonary malignancy, pleural plaques, pneumothorax, or pulmonary embolus). These conditions were confirmed through imaging, such as radiographs or computed tomography, performed in the BAM hospital before admission to the HAH program.

**Figure 1:**
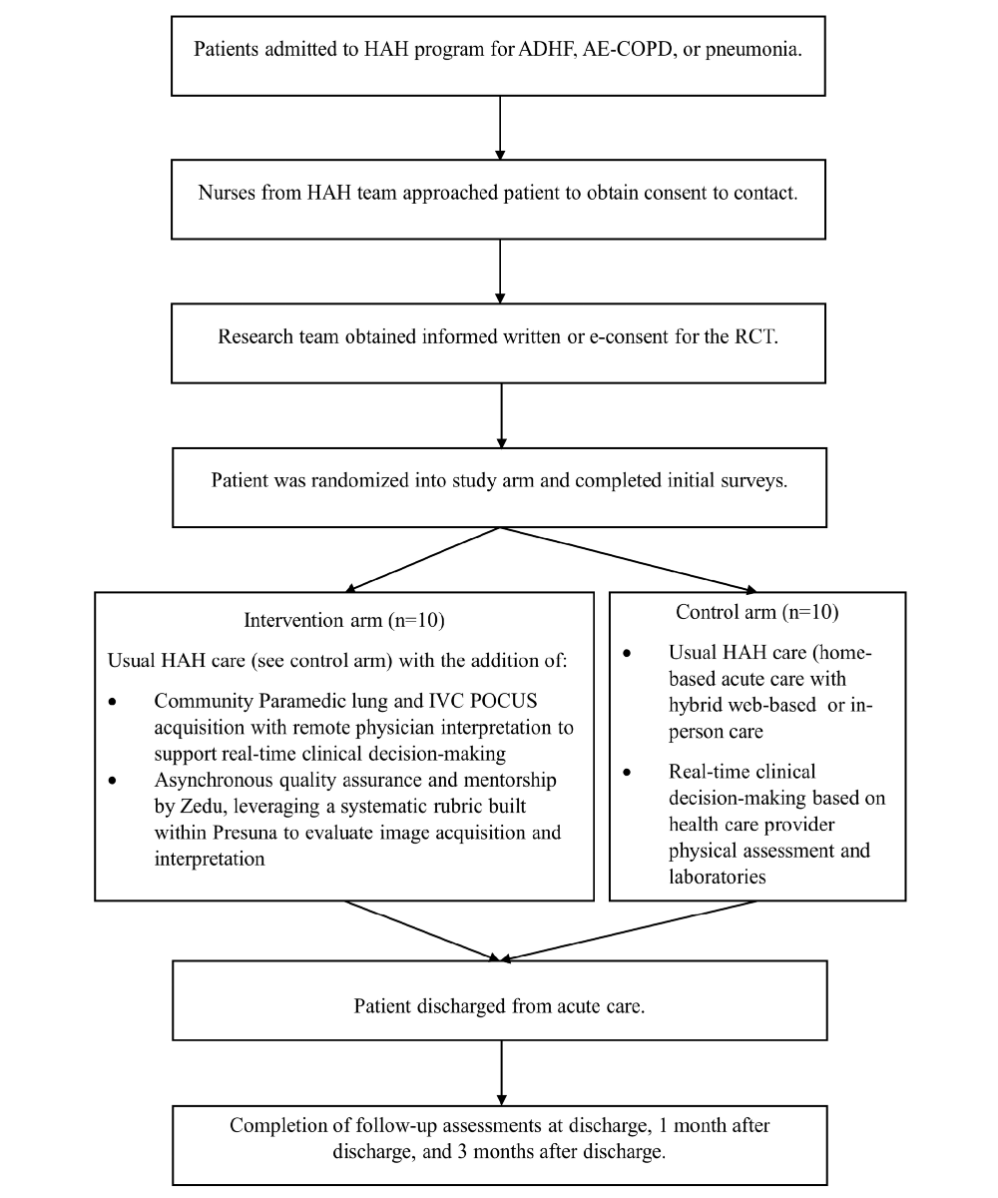
Randomization and study protocol. ADHF: acute decompensated heart failure; AE-COPD: acute exacerbation of chronic obstructive pulmonary disease; HAH: hospital at home; IVC: inferior vena cava; POCUS: point-of-care ultrasound; RCT: randomized controlled trial.

Enrolled study participants were randomized using REDCap’s randomization function to either the intervention arm (HAH care enhanced with serial POCUS assessment) or the control arm (usual HAH care; Figure 1).

### Health Care Provider Recruitment

HAH Health care providers were recruited based on an expressed willingness to participate in this study after being provided with information about the study’s aims and methods. Study staff then obtained informed consent via email or in person. When a physician did not want to participate in the study, 2 physicians from the research team who regularly attended HAH assumed care of study patients for the weeks in which there was a nonparticipating physician.

In preparation for the RCT, we previously trained 6 CPs in POCUS image acquisition for lung and IVC using a hybrid protocol, consisting of in-person didactic lectures and bedside teaching, online modules from the Canadian Point of Care Ultrasound Society [32], and asynchronous mentorship. The latter was used to evaluate the quality and clinical usability of participants’ POCUS images based on a rubric developed in consultation with POCUS experts.

**Figure 2:**
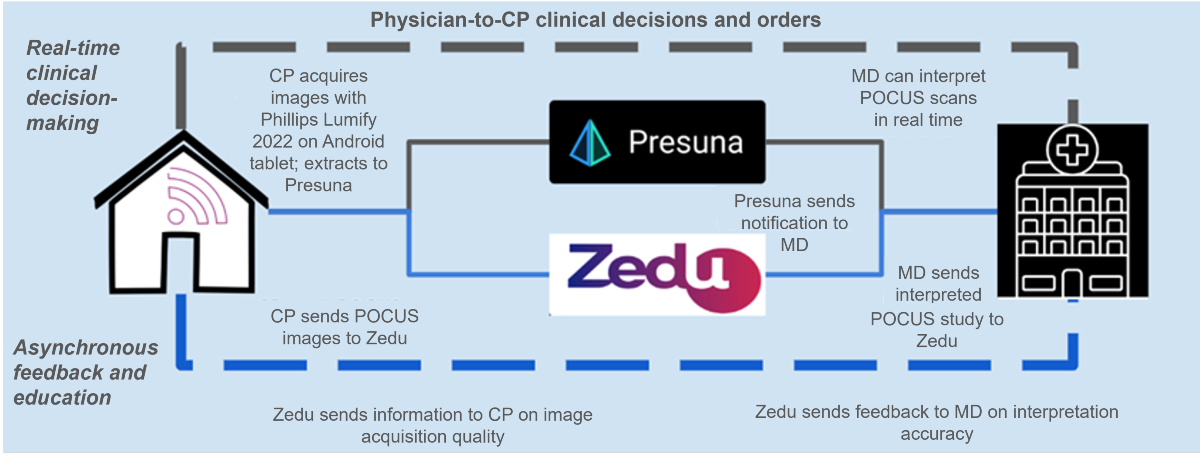
Process for remote acquisition, interpretation, and real-time learning of inferior vena cava (IVC) and lung point-of-care ultrasound (POCUS). CP: community paramedic; MD: doctor of medicine.

There were 2 groups of physician participants, a mix of non-POCUS experts and POCUS experts who regularly rotate through the service. Those defined as POCUS experts are physicians certified in POCUS use and capable of providing consultative support to other clinicians or trainees. These individuals required only a brief orientation to the cloud-based application used for image interpretation, as they were already proficient in POCUS use. Initially, we intended to train non-POCUS expert physicians in acquiring and interpreting lung and IVC POCUS using the same training program described earlier for CPs. However, the post–COVID-19 pandemic challenges of high patient volumes, multiple competing priorities, and physician burnout made it infeasible for physicians to complete the training. Consequently, we pivoted to train 2 physicians from the research team to be able to provide real-time POCUS interpretation support for non-POCUS expert physicians (Figure 2). While certain physicians leveraged this support and the rubric on the cloud-based application to aid them in learning about POCUS, this was not common and was not evaluated in this study.

### Randomization and Allocation Concealment

Randomization through the REDCap functionality allocated participants to their respective study arms. Data analysts, including economists, are blinded to study allocation. The master list of the clinical and study codes will be kept in an encrypted file on the University of Calgary server and managed by the research study team.

### Intervention

Patients randomized to the interventional study arm received serial lung and IVC POCUS assessments throughout their admission to HAH. These images were acquired by CPs in patients’ homes and transmitted to HAH physicians via Presuna, a cloud-based application. Presuna is a versatile software platform that allows ultrasonography programs to grow and maintain ultrasonography capacity through both in-person and online ultrasonography assessments and analytics. Key features include structured digital logbooks for supervised, on-site scan capture; flexible assessment templates that can be tailored to individual program objectives; secure link sharing for soliciting second opinions from expert reviewers; and built-in user surveys timed to critical workflow events (eg, immediately after scan upload or interpretation). The platform’s analytics dashboards provide real-time insights at both individual and cohort levels, enabling programs to monitor procedural volumes, acquisition quality, and interpretive accuracy and to benchmark performance against competency thresholds.

During development, Presuna followed a user-centered, iterative design process with ultrasonography experts, CPs, and physicians to ensure its workflows and interfaces met real-world needs. Enhancements were developed throughout the pilot around the asynchronous online assessments, assessment templates, and analytics.

In pilot testing, Presuna was deployed in collaboration with Zedu Ultrasound Training Solutions and Alberta Health Services’ Mobile Integrated Healthcare program for community paramedicine. During the pilot, CPs performed POCUS in patient homes, uploading studies via digital tablets to Presuna. Physicians used the platform’s customizable assessment templates and secure link sharing to interpret scans remotely and solicit second opinions from ultrasonography experts. Every study then underwent centralized quality assurance review by Zedu educators, leveraging built-in user surveys triggered immediately after uploads and interpretations to capture usability and contextual research data in real time.

The built-in user survey was structured and quantitative. It was administered to assess the implementation and clinical integration of POCUS. The survey included closed-ended questions using Likert scales and multiple-choice formats to evaluate technical issues affecting workflow, perceived quality of acquired scans, and health care provider confidence in scan acquisition. Additional items assessed interpretation consistency with expert reviewers for lung IVC scans, changes in interpretation following expert consultation, confidence in scan interpretation, and the perceived usefulness of POCUS findings for clinical decision-making and patient management. Responses were collected after each POCUS encounter to capture real-time health care provider experience.

Throughout the pilot, Presuna’s competency analytics dashboards were used to track acquisition quality and interpretive accuracy at both the individual and cohort levels. These analytics informed when CPs and physicians reached predefined competency thresholds that allowed them to more safely participate in the RCT phase of the pilot.

Overall, Presuna allowed for the extraction and secure sharing of the images captured by the handheld POCUS. CPs used a Phillips Lumify C5-2 curved array ultrasound transducer connected to a Samsung tablet to extract the images from the Lumify app and upload them to the Presuna application (which is encrypted). Once on Presuna, the images were instantaneously available for the HAH physicians to interpret, either on a hospital computer or their personal cell phones, facilitating immediate decision-making by remote physicians (Figure 2).

**Figure 3:**
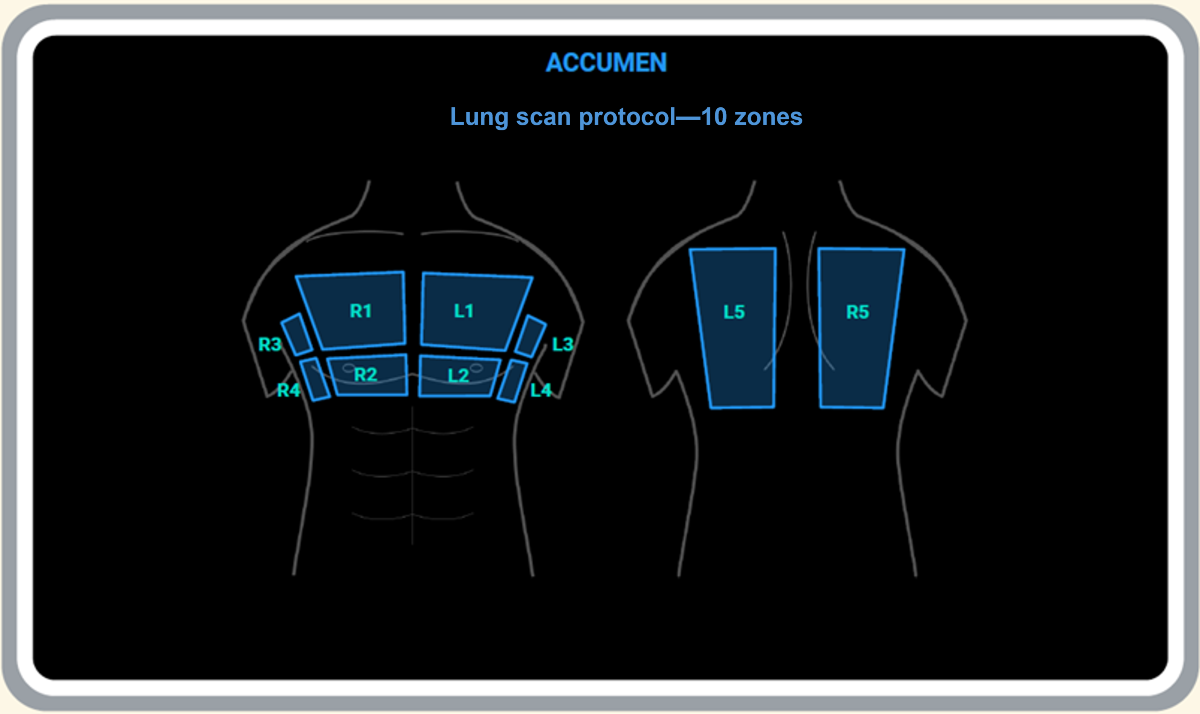
Lung zones. L1: left upper anterior zone; L2: left middle anterior zone; L3: left upper lateral zone; L4: left lower lateral zone; L5: left upper posterior zone; R1: right upper anterior zone; R2: right middle anterior zone; R3: right upper lateral zone; R4: right lower lateral zone; R5: right upper posterior zone.

For each study participant in the intervention arm, CPs acquired images for a minimum of 8 out of 10 lung zones (Figure 3). They attempted to acquire 2 views of IVC (longitudinal and transverse) with measurements at end inspiration and expiration to derive the IVC collapsibility index.

### Ethical Considerations

#### Human Participant Ethics Review Approvals or Exemptions

This study was approved by the Conjoint Human Research Ethics Board at the University of Calgary (REB22-0434; latest protocol amendment version: 13.0; issue date: March 27, 2025). This study is registered as a clinical trial on ClinicalTrials.gov (NCT05423652). Ethics approval covered all aspects of the study, including patient identification, consent procedures, and data collection.

#### Informed Consent

As described in the Patient Recruitment section, patients admitted to the HAH program with ADHF, AE-COPD, or pneumonia were identified as potentially eligible either by HAH NNs or through chart screening by the research team under a waiver of consent. Eligible patients were offered the opportunity to be contacted by the research team to learn more about the study. Following initial contact, consenting patients were enrolled by the research team, who obtained written or verbal informed consent via REDCap or email.

#### Privacy and Confidentiality

Participant privacy and confidentiality were maintained throughout the study. Each participant was assigned an anonymous study number, which was the only identifier for POCUS scans on Presuna and used during the data analysis. Any data containing personal identifiers were stored securely in locked cabinets or password-protected digital files accessible only to the research team. All reported data were aggregated to prevent the identification of individual participants.

#### Compensation Details

No compensation was provided to research participants.

### Data Collection

All data on Presuna are encrypted in transit with modern transport layer security and while at rest (storage) with modern Advanced Encryption Standard 256-bit encryption. Patient study IDs were used as the only method of patient identification. Consequently, no identifiable patient information was stored on Presuna. Furthermore, Presuna uses the “least access principle,” so health care providers could only access the data needed to perform their workflows and inform their decisions. In addition, all practitioners accessing Presuna had their codes to protect them as health care provider participants in the study and reduce the identifiable linkages of patients with their health care providers.

All other study data were collected and managed using REDCap electronic data capture tools hosted at the Centre for Health Informatics, Cumming School of Medicine, University of Calgary, Calgary, Alberta, Canada [33,34]. REDCap is a secure, web-based application designed to support data capture for research studies providing an intuitive interface for validated data capture; audit trails for tracking data manipulation and export procedures; automated export procedures for seamless data downloads to standard statistical packages; and procedures for data integration and interoperability with external sources. The principal investigator and the research team members involved in data analysis will be given access to the cleaned datasets. To ensure confidentiality, data dispersed to research team members will be blinded to any identifying participant information.

### Study Outcomes

#### Overview

The demographic characteristics collected in this study includes date of birth, age, sex or gender, comorbid diagnoses, Charlson Comorbidity Index, education level, employment status, race, smoking history, medication history, home medications, goals-of-care status, and overall functional status. This serves to stratify end points based on potentially extraneous variables, thus minimizing their confounding effect in the final analysis. Study outcomes are intended to capture the most clinically significant causes of patient morbidity and mortality.

Data were collected to evaluate the added clinical value of incorporating POCUS for lung and IVC into our HAH care in terms of its impact on clinical outcomes, feasibility, and sustainability.

#### Primary Outcome

The primary outcome is defined as LOS for the index HAH admission, measured from the date of admission until discharge, based on ADHF studies showing decreased LOS in BAM inpatient care [12]. As the program has an acute (hospital substitution) and subacute arm (after acute care), LOS on each arm will also be reported.

#### Secondary Outcomes

Textbox 1 summarizes the metrics according to the quadruple aim framework, which guided our evaluation of clinical outcomes, patient- and clinician-reported metrics, as well as health care system sustainability outcomes.

**Study metrics by category.**

**Patient outcomes**

- Health-related quality of life (EQ-5D-5L) at the time of randomization, within 1 week following hospital at home (HAH) discharge, and at 3 months after discharge, along with other objective metrics, will be assessed.
- Disease-related metrics for pneumonia are as follows:
  Time to resolution of infection (return to baseline oxygen saturation level) and normalization of white blood cell count
  For intervention-group participants, the normalization of pathological B-lines due to consolidative pneumonia
  Change in hemoglobin and white blood cell count during the enrollment period
- Disease-related metrics for acute decompensated heart failure are as follows:
  Time to decongestion (absence of crackles on lung auscultation and return to baseline oxygen saturation levels)
  For the intervention group, the absence of pathological B-lines or pleural effusions on ultrasound
  Change in N-terminal pro–B-type natriuretic peptide, troponin, creatinine, and estimated glomerular filtration rate during the enrollment period
- Disease-related metrics for acute exacerbation of chronic obstructive pulmonary disease are as follows:
  Time to reduction in dynamic hyperinflation defined as the absence of wheeze and return to baseline respiratory effort and oxygen levels
  Absence of new pulmonary findings because point-of-care ultrasound (POCUS) monitoring in this population was intended to proactively identify new findings (eg, pneumonia) that could complicate the acute exacerbation of chronic obstructive pulmonary disease
  Change in hemoglobin and white blood cell count during the enrollment period
- Imaging modalities performed as a function of routine clinical care were used to support diagnosis before enrollment as well as during clinical status changes during the index HAH admission, including chest X-rays, computed tomography, and ventilation-perfusion scans to rule out pulmonary embolism.
- Medication burden was assessed at the time of randomization, until 1 week following discharge from HAH.
- Clinical status changes from the date of randomization until discharge from the index HAH admission were assessed. Changes in patient status were evaluated in terms of their severity and impact. Escalation of care is defined as the incidence ratio of transfer back to a brick-and-mortar hospital ward from HAH for a reason concerning clinical stability.
- Adverse events measured in standard monitoring of the program include new venous thromboembolism, falls, infection, and mortality.

**Patient or caregiver experience**

- Self-reported patient experience surveys after discharge from the index HAH admission, with quantitative Likert scales and free text options (refer to Multimedia Appendices 1-3) were conducted.

**Health care provider experience**

- Semiquantitative surveys of nurse practitioners, community paramedics (CPs), and physicians providing care to patients in the study were conducted. Surveys were administered to inquire into health care providers’ experience with the HAH model and various technologies used over the course of its duration (digital remote patient monitoring, videoconferencing, as well as POCUS Presuna for the intervention arm). Participants were asked to score their responses on a Likert scale, allowing entries in the form of strongly disagree, disagree, neither agree nor disagree, and strongly agree.
- We embedded questions after each encounter in a clinical survey on Presuna for CPs and physicians to complete regarding their confidence in acquiring and interpreting images (both in terms of their own confidence and the image quality), respectively. We also asked questions to determine the usability and clinical usefulness of POCUS for each encounter.

**Health care use and cost**

- Length of stay for the index HAH admission (refer to the aforementioned information) will be calculated.
- Readmission rate (7, 30, and 90 days after discharge from HAH) will be calculated.
- Admission to facility living, including long-term care or supportive living, will be assessed up to 12 months following discharge from the index HAH admission.
- Time per CP visit will be calculated.
- Cost comparison of the cost of CP, physician, nursing services, medications, and diagnostic testing will be done. We will leverage Alberta’s health data repositories, including physician claims; National Ambulatory Reporting System; Discharge Abstracts Database; and Data Integration, Measurement and Reporting, as well as Alberta Health Services’ cost estimates.

#### Statistical Analysis

The study by Öhman et al [35] showed a 46% reduction in hospital LOS in the standard care group versus the treatment arm that used cardiothoracic ultrasound to guide clinical decision-making for inpatient care (mean 6.85, SD 4.22 days versus mean 3.72, SD 2.02 days). For our study, we will use a conservative 30% reduction in LOS on HAH for the intervention arm versus the control arm, powered at 80% with an α level of .05. In designing the study, we estimated that we would require a sample size of 22 patients per study arm within the time frame of this project.

The analysis and reporting of this trial will be undertaken in accordance with CONSORT (Consolidated Standards of Reporting Trials) guidelines [36]. We will conduct an intention-to-treat analysis, including all randomized patients who receive at least 1 study intervention or control procedure. We will also conduct a per-protocol analysis [37] based on patients who complete the study without major protocol deviations affecting primary end points and a safety analysis of all randomized patients who receive any study-related care.

For the primary outcome of HAH LOS, we will conduct a 2-sample 2-tailed *t* test (if normally distributed) or a Mann-Whitney *U* test (if nonnormally distributed) between the intervention and control groups. We will report the effect size with 95% CIs alongside the *P* values. Given the small sample size, no subgroup analyses will be conducted.

An analysis of resource use and costs will be conducted to inform the economic impact of HAH through an economic evaluation and budget impact analysis. We will use the data gathered by the study, supplemented with data from health care administrative databases, for this analysis. The implementation of HAH is anticipated to yield improvements in both system efficiency and patient outcomes, and the economic evaluation will quantify the costs and benefits associated with HAH compared with conventional care models.

For analysis of secondary and economic outcomes, we will report outcomes, *P* values, and CIs, but formal hypothesis testing will be avoided, given the exploratory nature of the analysis. However, any notable imbalances will be described and considered in the interpretation of results. For analysis of the net resource effectiveness, economic data will be analyzed via the headroom model, which is a method that quantifies the greatest cost at which an intervention would continue to demonstrate a net economic benefit. In this study, the headroom analysis will compare the control group, defined earlier as standard practice HAH care, with the POCUS or Presuna (intervention) group in patients with ADHF, AE-COPD, and pneumonia. Secondary patient outcomes will be analyzed via a 2 tailed *t* test comparing differences in biomarker or imaging values between control and intervention groups, set to the same power of 0.80 and an α level of .05.

#### Quality Assurance, Adverse Event Monitoring, and Steering Committee

Several measures were enacted to improve adherence to intervention protocols and ensure patient safety. Quality assurance of randomly selected scans was performed by the research team in consultation with a POCUS expert physician. This involved asynchronous mentorship leveraging the Presuna platform and Zedu Ultrasound Training Solutions. We created a POCUS acquisition or interpretation evaluation rubric to evaluate the presence of proper anatomical landmarks, image quality, and systematic analysis of the scans to identify the presence or absence of pathological findings.

A structured, modular rubric was used within the Presuna platform to assess both the quality of ultrasound image acquisition and the accuracy of scan interpretation. The rubric was designed to separate scan-level observations from examination-level conclusions and was tailored to the unique requirements of different examination types. For lung ultrasound, physician reviewers documented specific scan findings such as A-lines, lung sliding, B-lines, and pleural effusion while also making global diagnostic determinations across the entire examination (eg, interstitial edema and pneumothorax). For IVC assessments, which require integration of multiple clips, the rubric focused on examination-level evaluation, including visual estimates of IVC size and collapsibility, and clinical estimation of central venous pressure. Quality assurance reviewers used a parallel rubric to evaluate acquisition quality, with domains covering technical parameters (eg, depth and gain), anatomical accuracy (eg, correct orientation and landmark visualization), and interpretability (eg, completeness, labeling, and diagnostic utility). All questions were formatted as categorical selections (eg, yes or no, multiple choice, and ordinal scales) to ensure consistency and enable quantitative tracking. The rubric was adaptable to site-specific protocols and levels of training, allowing for reliable, scalable assessment across educational and clinical implementations. Adverse events were stratified under frequency and severity of venous thromboembolism, new infections (focal or systemic), falls, delirium, and medication-related reactions, or other clinically significant events. They were recorded from the date of randomization up to discharge from HAH. Mortality outcomes will be stratified under time to death and cause of death from date of randomization until participant death up to 1 year after discharge.

The data and safety monitoring board reviewed potential adverse events during the study. The data and safety monitoring board was composed of 2 GIM specialists who worked on HAH and were trained in POCUS for this study and 2 expert POCUS physicians. These 2 expert POCUS physicians (a GIM physician who works at a different hospital and a thoracic surgeon) have extensive experience in the design and deployment of ultrasound-based training curricula.

Finally, a steering committee for the project met monthly to discuss project operations and updates as well as provide direction and guidance to the research team. This committee consisted of the research team and coinvestigators, a human factor evaluation team, the CP manager, POCUS experts, advisors, and a health economics analyst.

## Results

Study funding began in April 2022, with data collection commencing in December 2023. Patient recruitment was finalized on December 31, 2024. This study includes a 3-month follow-up for major outcomes and a 1-year follow-up for long-term health care use, including admissions to long-term care. A total of 20 patients were enrolled (intervention group: n=10, 50%; control group: n=10, 50%).

Preliminary findings were presented in poster format at the World Hospital at Home Congress (March 2025). Initial results of an analysis of patient and health care provider experience highlighted the feasibility and potential benefits of remotely acquired POCUS imaging in HAH. Full data analysis is in progress, with results to be disseminated through journal publication and future scientific conferences.

## Discussion

### Anticipated Findings

This study aims to evaluate the feasibility and clinical impact of POCUS for lung and IVC assessments acquired by CPs in the HAH setting. We hypothesized that remote POCUS would enhance clinical decision-making and improve outcomes for patients with ADHF, AE-COPD, and pneumonia. Preliminary findings suggest that remote POCUS is feasible and may offer clinical value in HAH, particularly in guiding volume status and respiratory assessments.

### Comparison With International Literature

This study builds on emerging evidence supporting the use of POCUS in home-based care. Previous studies have demonstrated the utility of lung ultrasound in diagnosing pneumonia and monitoring pulmonary congestion in patients with heart failure, but few have explored its integration into online care models. Our approach, leveraging remote guidance and interpretation, addresses a critical gap in the literature by enabling real-time image interpretation to support clinical decision-making without requiring the physical presence of a sonographer or physician, which may be particularly beneficial in resource-limited or geographically dispersed settings.

### Strengths and Limitations

The strengths of this study include its randomized controlled design, multisite implementation, and focus on real-world feasibility. The integration of health care provider feedback and patient-reported outcomes adds depth to the evaluation, offering a more holistic understanding of remote POCUS in HAH.

Despite these promising insights, several limitations must be acknowledged. The most substantial limitation is the reduced sample size; although our power calculations indicated a need for 22 patients per arm, only 10 patients were recruited per group due to shifts in HAH admission patterns after the COVID-19 pandemic. This resulted in lower numbers of patients with ADHF, AE-COPD, and pneumonia than expected. While we implemented multiple strategies to increase recruitment, including adding the second site and obtaining a no-cost extension from the funder, we were only able to recruit 10 patients per study arm. This will limit our ability to detect statistically significant differences in the primary outcome and some secondary outcomes. However, the rich qualitative and semiquantitative data collected will still provide valuable insights into patient and health care provider experiences, workflow integration, and perceived utility of remote POCUS.

### Future Directions

Future directions include expanding the study to additional sites with higher volumes of target diagnoses, refining recruitment strategies, and exploring automated image interpretation tools to further streamline remote assessments. A dissemination plan is underway, with preliminary findings already presented at the World Hospital at Home Congress (March 2025). Full results of the clinical trial are currently being analyzed and will be submitted for peer-reviewed publication. Once the analysis is completed, we intend to present the finalized clinical trial results at upcoming conferences.

### Conclusions

This study represents the first RCT investigating remotely acquired POCUS for lung and IVC real-time decision-making in HAH care. While limited by sample size, preliminary findings support the feasibility and potential clinical value of remote POCUS in guiding care for patients with ADHF, AE-COPD, and pneumonia in the HAH setting. The integration of POCUS into HAH may enhance diagnostic accuracy, inform treatment decisions, and improve patient and health care provider experiences. These insights will inform future research and may contribute to the development of guidelines for incorporating POCUS into home-based acute care models.

## Appendix

Multimedia Appendix 1 Health care provider pre-post training survey.

Multimedia Appendix 2 Patient experience survey.

Multimedia Appendix 3 Health care provider experience survey.

## Abbreviations

ADHF: acute decompensated heart failure
AE-COPD: acute exacerbation of chronic obstructive pulmonary disease
BAM: brick-and-mortar
CONSORT: Consolidated Standards of Reporting Trials
CP: community paramedic
GIM: general internal medicine
HAH: hospital at home
IVC: inferior vena cava
NN: nurse navigator
POCUS: point-of-care ultrasound
RCT: randomized controlled trial
REDCap: Research Electronic Data Capture
