# Supplementary material for: Aiding Chronic Obstructive Pulmonary Disease and Congestive Heart Failure Ultrasound-Guided Management Through Enhanced Point-of-Care Ultrasound (ACCUMEN-POCUS): Protocol for a Randomized Controlled Trial
Source: JMIR Res Protoc. 2025 Sep 23;14:e76186. doi: 10.2196/76186 (PMC12504898; doi:10.2196/76186)
Supplement: Multimedia Appendix 1 [file resprot_v14i1e76186_app1.pdf]

PRE/POST PROVIDER SURVEY

STUDY TITLE: Aiding COPD and CHF Ultrasound-guided Management through ENhanced Point Of Care UltraSound (ACCUMEN-POCUS

Please identify your level of comfort with the following with (1) being “not at all” and (5) being “completely”

	**1 Not at all**	**2 Very little**	**3 Neutral**	**4 Quite a bit**	**5 Competely**
I feel comfortable acquiring lung ultrasound images					
I feel comfortable recognizing B-lines					
I feel comfortable recognizing A-lines					
I feel comfortable recognizing the spine sign					
I feel comfortable recognizing the diaphragm					
I feel comfortable recognizing lung sliding					
I understand how to use the settings on the ultrasound machine to optimize image acquisition (eg: depth, gain, saving images etc)					
I am confident that I am able to identify and understand artifacts when acquiring images					
I am confident that I am able to identify and understand artifacts when interpreting images.					
I am confident in my ability to integrate lung ultrasound with my clinical assessment					
I am confident in my ability to incorporate lung ultrasound into my clinical decision-making (for physicians only)					

This is a Multimedia Appendix to a full manuscript published in the J Med Internet Res. For full copyright and citation information see http://dx.doi.org/10.2196/76186
