# Supplementary material for: Aiding Chronic Obstructive Pulmonary Disease and Congestive Heart Failure Ultrasound-Guided Management Through Enhanced Point-of-Care Ultrasound (ACCUMEN-POCUS): Protocol for a Randomized Controlled Trial
Source: JMIR Res Protoc. 2025 Sep 23;14:e76186. doi: 10.2196/76186 (PMC12504898; doi:10.2196/76186)
Supplement: Multimedia Appendix 2 [file resprot_v14i1e76186_app2.pdf]

ACCUMEN-PRESUNA PATIENT EXPERIENCE SURVEY

Study ID:______________________________

Date: ________________________________

This survey is part of the research study for Complex Care Hub (CCH) patients with heart and lung conditions to compare usual CCH care with CCH care enhanced by point of care lung ultrasound. Your answers will be kept confidential.

Experience with Care					
	Definitely	Somewhat	Unsure	No	Not applicable
1. Did you understand how the healthcare team would provide you care?[1,2](					
2. Once you arrived home, did you understand how to contact the healthcare team for support?[1,2]					
3. When you had questions for your healthcare team, did you get answers you could understand? [Error! Bookmark not defined.,3]					
4. Did a member of your healthcare team explain the purpose of new medications in a way you could understand?[Error! Bookmark not defined.,Error! Bookmark not defined.]					
5. Did a member of your healthcare team tell you about new medication side effects to watch for while at home? [Error! Bookmark not defined.,Error! Bookmark not defined.]					
6. If you expressed any fears or anxieties about your condition or treatment, did someone from your healthcare team discuss them with you?[Error! Bookmark not defined.,Error! Bookmark not defined.]					
7. Did a member of your healthcare team tell you about the warning signs regarding your illness or treatment to watch for while at home? [Error! Bookmark not defined.,Error! Bookmark not defined.]					
8. Were you involved in decisions made about your care and treatment to the level you wanted? [Error! Bookmark not defined.,Error! Bookmark not defined.]					
9. Did doctors, nurses, and other members on your healthcare team seem informed and up to date about your care?[1,4]					
10. While you received care from Complex Care Hub, did the healthcare team respond to your questions or concerns in a timely manner?[1]					

ACCUMEN-PRESUNA PATIENT EXPERIENCE SURVEY

11. Did you feel your care goals were included in your treatment as a Complex Care Hub patient?[1,2]					
**Experience with Care (continued…)**					
	Yes, definitely	Yes, to some extent	No	Unsure	Not applicable
12. Did you feel you were treated with respect and dignity while you received care from the Complex Care Hub?[1,4]					
13. Did the Complex Care Hub help you to maintain or regain your function and independence?[1,2]					
14. Did you ever feel concerned about your safety during the course of your care at home?[1,2]					
15. Would you recommend the Complex Care Hub to your family and friends? [1,2,5]					
16. Did the health care team prepare you to manage your condition once you no longer required Complex Care Hub (CCH)care?[1,2]					
Follow-up to question 16 (select any that apply)	More education	More technology	More time on the program	Extra services at home	I did not need anything more from CCH
17. Which of the following would have helped you be more prepared to manage your condition after leaving the Complex Care Hub?[6]					

Please share any additional comments about your care in the Complex Care Hub:

Types of technology assisted visits					
Choose all that apply:	Virtual consults (video/phone) between Community Paramedic & Doctor	Virtual video visits with Doctor	Virtual phone visits with doctor	Remote patient monitor (vital signs)	Point of care ultrasound
18. Which technology and visits did you have while you were on the Complex Care Hub (CCH)?[2]					
19. Please check all the types of technology-assisted visits you felt enhanced your care on the Complex Care Hub?					
20. Please check any of the types of technology-assisted visits you felt were not useful for your care on the Complex Care Hub?					
21. Please check any visits in which your providers or you had technical issues that impacted your care					
Please add any additional comments on the technology used for your care:					

Impact of technology on care						
Please read these statements and select the answer that best reflects how you feel	Strongly Disagree	Disagree	Neutral	Agree	Strongly Agree	Not applicable
22. Virtual visits on CCH improved my access to healthcare services.[7]						
23. My healthcare needs were met through virtual visits.[7]						

24. The use of CloudDx (remote patient monitoring) allowed me to have control over my condition.						
**Impact of technology on care (cont. …)**						
Please read these statements and select the answer that best reflects how you feel	Strongly Disagree	Disagree	Neutral	Agree	Strongly Agree	Not applicable
25. My overall experience with virtual care was the same as an in-person visit.[7] (Virtual care: care that has at least one or more components done remotely e.g., video, telephone, online)						
26. I had enough access to the doctor while on the Complex Care Hub.						
27. Point of care ultrasound helped my care team assess my condition						
28. Point of care ultrasound helped my care team make more informed decisions about my care plan						
29. I felt comfortable while point of care ultrasound was being performed on my body.						
30. The addition of point of care ultrasound helped me have more control over my condition.						
31. I would like to keep receiving point of care ultrasound to help better manage my condition(s).						
Please add any additional comments on the technology used while on CCH:						

**Overall Satisfaction**	Very Dissatisfied	Dissatisfied	Neutral	Satisfied	Very Satisfied
32. How satisfied are you with the way the health care team worked together?Error! Bookmark not defined.					
33. Overall, how satisfied are you with the care you received from CCH?[2]					

Helpfulness of Program										
34. Overall, do you feel you were helped by the Complex Care Hub? Please answer on a scale where 0 is "not helped at all" and 10 is "helped completely.[1,4]										
Not helped at all										Helped completely
**0**	**1**	**2**	**3**	**4**	**5**	**6**	**7**	**8**	**9**	**10**

Alternatives to Complex Care Hub								
	In-person visit with regular health care provider	Visited a walk-in clinic	Visited an emergency department	Scheduled an appointment with another healthcare provider	Self-managed my health care issue at home	Would have stayed in hospital	Done nothing. I would not have sought care	Other
What would you have done if the virtual care option was not available?								
If you selected other, please describe what you would have done.								

Extra comments
Is there anything you would like to share about your Complex Care Hub experience?[4]
Is there anything that could be done to better meet your needs?[4]

References

[1] Health Quality Council of Alberta (2018). The Alberta Quality Matrix for Health. http://hqcasurveys.hqca.ca/redcap/surveys/index.php?s=WDCNDYDPEL

[2] Wodinski, L., Gibbons-Reid, V., Fraser, A., Fikry, M., Reynolds, C., & El-Hajj, J. (2020). Provincial Virtual Hospital Patient Survey 2020-2021. Health Systems Evaluation and Evidence. Alberta Health Services.

- Note: This material is intended for general information only and is provided on an "as is", "where is" basis. Although reasonable efforts were made to confirm the accuracy of the information, Alberta Health Services does not make any representation or warranty, express, implied or statutory, as to the accuracy, reliability, completeness, applicability or fitness for a particular purpose of such information. This material is not a substitute for the advice of a qualified health professional. Alberta Health Services expressly disclaims all liability for the use of these materials, and for any claims, actions, demands or suits arising from such use. HSEE created and customized the patient survey from multiple validated tools (e.g., PPE-15, CPES, HCAHPS, HQCA PCS, TUQ-10) to capture the context of a virtual hospital.

[3] Jenkinson, C., Coulter, A., & Bruster, S. (2002). Picker Patient Experience Questionnaire: development and validation using data from in-patient surveys in five countries. International Journal for Quality in Health Care, 14(5), 253-358. https://doi.org/10.1093/intqhc/14.5/353

[4] Canadian Institute of Health Information (2019). The Canadian Patient Experiences Survey on Inpatient Care (CPES-IC). https://www.cihi.ca/en/patient-experience

[5] Centers for Medicare & Medicaid Services (March 2020). Hospital Consumer Assessment of Healthcare Providers and Systems (HCA HPS). https://hcahpsonline.org/globalassets/hcahps/survey-instruments/mail/effective-july-1-2020-and-forward-discharges/2020_survey-instruments_english_mail.pdf

[6] Citizen SUS is in reference to the Virtual Care System and Use Survey (Citizens) by Canada Health Infoway: https://www.infoway-inforoute.ca/en/component/edocman/3862-virtual-care-system-and-use-survey-template-citizens/view-document?Itemid=0

[7] Parmanto, B., Lewis Jr, L., Graham, K., & Bertolet, M. (2016). Development of the telehealth usability questionnaire (TUQ). International Journal of Telerehabilitation, 8(1), 3-10. https://.doi.org/10.5195%2Fijt.2016.6196

This is a Multimedia Appendix to a full manuscript published in the J Med Internet Res. For full copyright and citation information see http://dx.doi.org/10.2196/76186
