# Supplementary material for: Aiding Chronic Obstructive Pulmonary Disease and Congestive Heart Failure Ultrasound-Guided Management Through Enhanced Point-of-Care Ultrasound (ACCUMEN-POCUS): Protocol for a Randomized Controlled Trial
Source: JMIR Res Protoc. 2025 Sep 23;14:e76186. doi: 10.2196/76186 (PMC12504898; doi:10.2196/76186)
Supplement: Multimedia Appendix 3 [file resprot_v14i1e76186_app3.pdf]

Alberta Health Services

UNIVERSITY OF CALGARY

ACCUMEN-PRESUNA PROVIDER EXPERIENCE SURVEY

Study ID:______________________________

Date:__________________________________

This survey is part of the research study for Complex Care Hub (CCH) patients with Congestive Heart Failure, COPD and pneumonia to compare usual CCH care with CCH care enhanced by point of care lung ultrasound (POCUS). Your answers will be kept confidential.

Types of technology assisted visits					
(check all that apply)	Virtual consults between providers	Virtual video visits with patients	Virtual phone visits with patients	Remote patient monitor (vital signs)	Point of Care Ultrasound (POCUS)
1. Which ***technology and visits*** did you use on the Complex Care Hub this past week?**Error! Bookmark not defined.**					
2. Please check any technology you felt **added value** to patient care					
3. Please check any technology you felt **detracted value** from patient care					
4. Please check any visits in which you had ***technical issues*** that impacted patient's care					
Please share any additional comments about the impact of technology on patient care:					

This is a Multimedia Appendix to a full manuscript published in the J Med Internet Res. For full copyright and citation information see http://dx.doi.org/10.2196/76186

Impact of technology on care and team relationships						
**Please read these statements and select the answer that best reflects how you feel**:	Strongly Disagree	Disagree	Neutral	Agree	Strongly Agree	Not applicable
5. The use of point of care ultrasound (POCUS) had a positive impact on the dynamic of the healthcare team						
6. The use of POCUS had a negative impact on the dynamics of the healthcare team						
7. The use of POCUS had a positive impact on the relationship with the patient(s)						
8. The use of POCUS had a negative impact on the relationship with the patient(s)						
9. Telehealth improved my ability to provide healthcare services.[1]						
10. My patients' healthcare needs were met using CloudDx.						
11. The use of CloudDx (remote patient monitoring) allowed me to support patients in self-management of their condition(s).						
12. Videoconferencing enabled me to connect with my patients.						
13. My overall experience with Telehealth was the same as an in-person visit.[2]						
14. POCUS helped the care team assess patient(s)' cardiopulmonary condition(s) more accurately						
15. POCUS helped the care team make more informed decisions about patient(s)' care plans						
16. I felt comfortable performing POCUS						

17. I felt comfortable using the images acquired remotely to make a diagnosis (for physicians only)						
18. It was easy to use POCUS in my clinical work						
19. It was easy to use PRESUNA in my clinical work						
20. The use of POCUS allowed me to support patients in self-management of their condition(s).						

In the following section, we are interested in your perception of factors that impacted the care of your patients while on this study. We are interested in your overall experience while on service in the past week.

	Intervention arm (CCH care + POCUS)	Control Arm (CCH care only)	They were the same
21. Patient clinical assessments were more time-intensive			
22. Clinical decision-making for the patients were more time intensive			
23. Documentation was more time intensive			
24. Quality of care was better			
25. Patient experience seemed better			
26. Ability to manage patients was easier			
27. Ability to discharge patients was easier			
28. Providing patient care was more interesting			
29. Accuracy of clinical assessments was better			
30. Treatment decisions were more proactive			

Helpfulness of Program

31. Overall, how helpful was the intervention arm (CCH + POCUS-PRESUNA) for the patients? Please answer on a scale where 0 is "not helped at all" and 10 is "helped completely.[3,4]

Not helped at all 0	---------- 1	2	3	4	5	6	7	8	9	Helped completely 10	Not Applicable

32. Overall, how helpful was the control arm (CCH usual care) for the patients? Please answer on a scale where 0 is “not helped at all” and 10 is “helped completely.Error! Bookmark not defined.,Error! Bookmark not defined.											
Not helped at all 0	---------- 1	2	3	4	5	6	7	8	9	Helped completely 10	Not Applicable

Overall Satisfaction

	Very Dissatisfied	Dissatisfied	Neutral	Satisfied	Very Satisfied	Not Applicable
33. How satisfied are you with the way the health care team worked together to provide care for CCH patients?[3]						
34. Overall, how satisfied are you with the care you provided on the Complex Care Hub?[5]						
35. How satisfied are you with the care provided to patients on the intervention arm (CCH care + POCUS)?						
36. How satisfied are you with the care provided to patients on the control arm (usual CCH care without POCUS)?						
37. How satisfied were you with using PRESUNA to capture POCUS findings over time?						
38. How satisfied were you with using Phillips Lumify POCUS device?						
**Comments** - please elaborate on areas where you were **dissatisfied:**						

**Comments** – please elaborate on areas where you were **satisfied:**				

**Recommendations**				
	Definitely	Somewhat	Unsure	No
39. Would you recommend using remotely acquired lung and IVC POCUS images to a colleague?				
40. Would you recommend using PRESUNA to a colleague?				

41. Is there anything you would like to share about your experience using POCUS and/or PRESUNA on CCH?	

References

[1] Parmanto, B., Lewis Jr, L., Graham, K., & Bertolet, M. (2016). Development of the telehealth usability questionnaire (TUQ). International Journal of Telerehabilitation, 8(1), 3-10. https://.doi.org/10.5195%2Fijt.2016.6196

[2] Centers for Medicare & Medicaid Services (March 2020). Hospital Consumer Assessment of Healthcare Providers and Systems (HCA HPS). https://hcahpsonline.org/globalassets/hcahps/survey-instruments/mail/effective-july-1-2020-and-forward-discharges/2020_survey-instruments_english_mail.pdf

[3] Health Quality Council of Alberta (2018). The Alberta Quality Matrix for Health. http://hqcasurveys.hqca.ca/redcap/surveys/index.php?s=WDCNDYDPEL

[4] Canadian Institute of Health Information (2019). The Canadian Patient Experiences Survey on Inpatient Care (CPES-IC). https://www.cihi.ca/en/patient-experience

[5] Wodinski, L., Gibbons-Reid, V., Fraser, A., Fikry, M., Reynolds, C., & El-Hajj, J. (2020). Provincial Virtual Hospital Patient Survey 2020-2021. Health Systems Evaluation and Evidence. Alberta Health Services.

- Note: This material is intended for general information only and is provided on an "as is", "where is" basis. Although reasonable efforts were made to confirm the accuracy of the information, Alberta Health Services does not make any representation or warranty, express, implied or statutory, as to the accuracy, reliability, completeness, applicability or fitness for a particular purpose of such information. This material is not a substitute for the advice of a qualified health professional. Alberta Health Services expressly disclaims all liability for the use of these materials, and for any claims, actions, demands or suits arising from such use. HSEE created and customized the patient survey from multiple validated tools (e.g., PPE-15, CPES, HCAHPS, HQCA PCS, TUQ-10) to capture the context of a virtual hospital.
